# Safety, feasibility, and tolerability of psilocybin in older adults with amnestic MCI: Preliminary data from a SV2a PET imaging study

**DOI:** 10.1002/alz70859_101101

**Published:** 2025-12-25

**Authors:** Danielle Bukovsky, Aron Amaev, Jianmeng Song, Edgardo Torres‐Carmona, Shannen Kyte, Fumihiko Ueno, Vincenzo Deluca, Christopher R Bowie, Alastair Flint, Ishrat Husain, Ariel Graff‐Guerrero, Philip Gerretsen

**Affiliations:** ^1^ Centre for Addiction and Mental Health, Toronto, ON Canada; ^2^ University of Toronto, Toronto, ON Canada; ^3^ Queen's University, Kingston, ON Canada; ^4^ Department of Psychiatry, Temerty Faculty of Medicine, University of Toronto, Toronto, ON Canada; ^5^ University Health Network, Toronto, ON Canada; ^6^ Toronto Dementia Research Alliance, Toronto, ON Canada

## Abstract

**Background:**

Amnestic mild cognitive impairment (aMCI) is characterized by synaptic loss and cognitive decline and is considered a precursor to Alzheimer’s Disease. Currently, there are no effective treatments for aMCI, and available treatments (i.e., cholinesterase inhibitors) do not appear to improve cognitive or functional outcomes. Psychedelics, including psilocybin, have regained interest for treatment of treatment‐resistant neuropsychiatric disorders. Research suggests psilocybin’s psychedelic and clinical effects may be due to interaction with the 5HT2A serotonin receptor (5HTA‐R) in the brain. Cognitive impairments, such as memory decline, have been associated with lower 5HT2A‐R density in the brain. Encouragingly, preclinical animal studies suggest that psilocybin may promote synaptogenesis in the brain, particularly in areas associated with learning and memory, likely through its interaction with the 5HT2A‐R. Psilocybin may represent a novel treatment to counter neurodegenerative progression and consequently improve cognitive outcomes in patients with aMCI.

**Method:**

The present double‐blind, placebo‐controlled randomized PET study will use the radioligand [^18^F]SynVesT to assess psilocybin’s effect on synaptic density in the hippocampus and prefrontal cortex of patients with aMCI, and whether these changes are associated with improved cognitive outcomes. aMCI participants and sex‐matched healthy controls will be randomized to receive either two doses of 25mg psilocybin or placebo one week apart. Participants will be monitored by a study physician and qualified therapist. PET scans are conducted pre‐ and one‐week post‐treatment. Clinical, safety, and neuropsychological assessments are done at baseline and 1‐, 4‐, and 12‐weeks post‐treatment. Safety measures include monitoring vital signs, suicidal ideation, and adverse events (AEs).

**Result:**

Pilot data from two aMCI participants (Male% = 50%, mean age = 71.0(±3.0), mean baseline MOCA = 22(±3.0)) and three healthy controls (Male% = 67%, mean age = 66.3(±5.1); mean baseline MOCA = 27.7(±1.5)) is available. All participants completed study procedures without issue. Psilocybin was well tolerated, with no unexpected or serious AEs. All expected AEs resolved without sequelae. Expected AEs included dizziness (n=4) and altered perception (n=3). Blinding remains in effect.

**Conclusion:**

The preliminary data suggest psilocybin is safe, well‐tolerated, and can be feasibly investigated in a supervised medical setting in older adults as a prospective treatment for aMCI.

